# Role of Endogenous Microbiota, Probiotics and Their Biological Products in Human Health 

**DOI:** 10.3390/nu5010058

**Published:** 2013-01-10

**Authors:** Gordon S. Howarth, Hanru Wang

**Affiliations:** 1 School of Animal and Veterinary Sciences, The University of Adelaide, Roseworthy Campus, South Australia 5371, Australia; E-Mail: hanru.wang@adelaide.edu.au; 2 Centre for Paediatric and Adolescent Gastroenterology, Children, Youth and Women’s Health Service, North Adelaide, South Australia 5006, Australia

**Keywords:** microbiota, microbiome, modifiers, probiotics, biofactors, probiotic-derived factors, probiotic mechanisms, supernatants, intestinal function

## Abstract

Although gut diseases such as inflammatory bowel disease, mucositis and the alimentary cancers share similar pathogenetic features, further investigation is required into new treatment modalities. An imbalance in the gut microbiota, breached gut integrity, bacterial invasion, increased cell apoptosis to proliferation ratio, inflammation and impaired immunity may all contribute to their pathogenesis. Probiotics are defined as live bacteria, which when administered in sufficient amounts, exert beneficial effects to the gastrointestinal tract. More recently, probiotic-derived factors including proteins and other molecules released from living probiotics, have also been shown to exert beneficial properties. In this review we address the potential for probiotics, with an emphasis on probiotic-derived factors, to reduce the severity of digestive diseases and further discuss the known mechanisms by which probiotics and probiotic-derived factors exert their physiological effects.

## 1. Introduction

The microbiota of the gastrointestinal tract in both humans and animals consists of microorganisms that flourish throughout the digestive tract. These living microorganisms form an enormous microbial community that includes both aerobic and anaerobic bacteria, as well as viruses, fungi and parasites [1]. The intestinal microbiota contributes to bowel health in the host by fermenting unused energy substrates, preventing growth of harmful pathogenic bacteria [2], and assisting the host immune system [3,4]. On the other hand, disordered and impaired microbiota communities are associated with conditions such as obesity [5], inflammatory bowel disease (IBD) [6], and critical illness [7]. Consequently, it is important to investigate therapeutic strategies aimed at manipulating the dysbiosis (disordered microbiobial community) influenced by stressors (disease or other factors). This could assist the endogenous microbiota in restoring a normal or more consolidated microbiota status. Ingested probiotics (health-promoting bacteria) or their released factors could alter the endogenous microbiota to achieve a beneficial balance in the bowel. Together the combined microbiota could reduce the severity of certain diseases while preventing others, ultimately improving human health. 

## 2. Mechanisms of Probiotic Action

Probiotics are broadly defined as living, non-pathogenic micro-organisms (usually bacteria) which, when administered in sufficient numbers, exert a positive influence on host health [8]. Only a minority of bacterial species meet this definition. Probiotics are primarily bacteria from the lactobacillus and bifidobacterium genera. However, *Lactococcus*, *Streptococcus*, and *Enterococcus* species, as well as some non-pathogenic strains of *Escherichia coli*, and certain bacilli and yeast strains may also act as probiotics [9]. The digestive tract is the natural endosymbiotic habitat for probiotic species. In humans, lactobacilli and bifidobacteria are commonly present in the vagina, and gastrointestinal tract [10] and bifidobacteria are also detectable in the mouth [11]. These probiotics act as commensal bacteria that contribute to the maintenance of a healthy intestinal environment. 

A number of animal and human studies have shown the beneficial effects of probiotics on health through maintenance of a healthy gut microbiota [12,13]. Some probiotics have the potential to reduce the severity of disease conditions ranging from the inflammatory bowel diseases, Crohn’s disease and ulcerative colitis, through to forms of cancer [14,15,16]. To this end, several probiotics have been developed for therapeutic purposes [17,18,19]. In this review, the mechanism of probiotics and the efficacy of probiotic-derived factors will be discussed. 

Mechanisms of probiotic action described to date include adhesion to the intestinal-lumen interface; competition with pathogens for receptor binding, nutrients and colonization; enhancement of mucosal barrier function; promotion of innate and adaptive immune responses; elaboration of bacteriocins; and modulation of cell kinetics, with further mechanisms of action likely to be identified [20]. 

### 2.1. Microbiota Composition

Probiotic administration has the potential to shift the microbiota composition from a pathogenic predominance towards a more beneficial microbiotic ecosystem [12,21]. However, due to the transient nature of probiotic colonization, of the microbial composition of the microbiota is likely to return to normal if further probiotic administration is ceased. Administration of the probiotic, *Clostridium butyricum*, to healthy broiler chickens over a period of 45 days significantly decreased the levels of *Escherichia coli*, *Salmonella* and *Clostridium perfringens*, and concurrently increased *Lactobacillus*, *Bifidobacterium* and *C. butyricum* levels in cecal material [12]. Furthermore, administration of probiotics (*Bifidobacterium* and *Lactobacillus*) for 7 days after elective laparoscopic radical surgery in colorectal cancer patients has been shown to (a) significantly restore intestinal microbiota composition in fecal samples, as measured by an increase in the levels of *Bifidobacterium*, *Lactobacillus*, and *Enterococcus*; (b) reduce counts of *Escherichia coli* and *Staphylococcus aureus* [21].

### 2.2. Adhesion as a Mean to Compete with Pathogens

Adhesion of bacteria to mucosal surfaces and epithelial cells is one of the key features of probiotic action [22]. Factors affecting probiotic adhesion include large surface proteins and mucus-binding proteins possessing regions homologous with binding domains of proteins such as lectins [23]. The lectin-like interactions often result from a number of mucus-binding proteins, such as MUB (a cell-surface protein produced by *Lactobacillus reuteri* encoded a gene from *L reuteri* 1063) [24]. These in turn promote interactions between the bacteria and host gut [23]. For example, *Lactococcus lactis* ssp. *lactis* BGKP1 has an auto-aggregation phenotype and mucin binding protein (MbpL) on its surface, which contributes to its adherence properties in the intestine *in vivo *and *in vitro* [22]. Moreover, MbpL on the surface of *Lactococcus lactis* ssp. *lactis* BGKP1 revealed greater binding affinity to gastric-type mucin proteins, such as MUC5AC [22]. In addition, a new surface mucin-binding protein, identified on the surface of *Bifidobacterium bifidum* species and referred to as “transaldolase”, has been reported to act as an important colonization factor, potentially assisting adhesion of *B. bifidum* to the gut [25]. 

Probiotic adhesion ability is also determined by pH and temperature levels during fermentation [22,26]. Deepika *et al.* have recently reported the adhesion abilities of probiotic *Lactobacillus rhamnosus *GG (LGG) to the intestinal mucosa with different fermentation conditions (pH and temperature), which consequently resulted in surface changes during this process [26]. Moreover, some adhesive proteins, such as α-enolase, elongation factor Tu (EF-Tu), glyceraldehyde-3-phosphate dehydrogenase (GAPDH), and GroES chaperonin, identified on the LGG surface, have been shown to play an important role in the adhesion of LGG to Caco-2 cells [26]. These binding proteins could also be expressed on other probiotic bacteria allowing them to participate in the process of probiotic adhesion in the human gastrointestinal tract [26]. Studies by Ramiah *et al.* [27] revealed a similar result. These investigators found that the surface-bound proteins, elongation factor Tu (EF-Tu), glyceraldehyde-3-phosphate dehydrogenase (GAPDH) and triosephosphate isomerase influenced adhesion of *L. plantarum* 423 to Caco-2 cells and prevented *Clostridium sporogenes* LMG 13570 and *Enterococcus faecalis* LMG 13566 from adhering to Caco-2 cells [27]. 

The ability of probiotics to inhibit the growth of pathogens has been widely investigated, and more recently, antimicrobial properties of probiotics have been described. *Lactobacillus fermentum* 202, *Lactobacillus gallinarum* 7001, *L. rhamnosus* 183, and *L. plantarum* L2-1 show great potential *in vitro* to inhibit certain intestinal pathogens including *Escherichia coli*, and *Salmonella* spp. [28]. *Escherichia coli* strain Nissle 1917 (EcN) has been reported to inhibit the growth of *E. coli*, *Salmonella enterica* serovar Typhimurium and *Listeria monocytogenes in vitro* [29]. Moreover, *Bifidobacterium breve* B632 (DSM 24706), B2274 (DSM 24707), B7840 (DSM 24708) and *Bifidobacterium longum* ssp. *longum* B1975 (DSM 24709) identified from infant feces also demonstrated antimicrobial activity against coliforms and other pathogenic bacteria, while displaying no cytotoxic activity against the infant gut epithelium. Consequently, these bacteria could be considered as potential probiotics for the treatment and prevention of enteric disorders in newborns [30]. Interestingly, *Lactobacillus salivarius* strain UCC118 triggered the induction of antimicrobial genes (CCL20, CXCL1 and CXCL2) in the Caco-2 cell line, providing evidence for the antimicrobial capacity of probiotics at the gene level [31].

Even though a wide variety of probiotics have demonstrated antimicrobial properties against certain pathogens, not all have been shown to be efficacious. For example, Parassol *et al.* reported that *Lactobacillus casei* DN-114 001 did not inhibit pathogenic EPEC strain adhesion to the human colon T84 cell line [32]. This is consistent with the action of the probiotic *Lactobacillus paracasei*, which demonstrated strong adhesion to Caco-2 cells but failed to prevent *L. monocytogenes* infection [33]. Regarding the latter issue, Koo *et al.* recently have raised a possible explanation. In their studies, pathogenic Listeria adhesion protein (LAP)-expressing recombinant probiotic *Lactobacillus paracasei* (Lbp^LAP^) successfully blocked adhesion, invasion, and translocation of *Listeria monocytogenes* in Caco-2 cells [33]. Interestingly, Lbp^LAP^ was also able to prevent *L. monocytogenes*-mediated cell damage, reduce *L. monocytogenes*-mediated cell cytotoxicity, and restore tight junction integrity [33]. Thus, the use of recombinant probiotics that express pathogenic adhesion proteins could be a new strategy to prevent pathogenic bacterial infection [33]. 

In summary, many probiotics have the ability to adhere to the intestinal-lumen interface; compete with pathogens, and perform receptor binding and colonization and hence be beneficial to the host. 

### 2.3. Maintenance of Intestinal Integrity

The integrity of the intestinal barrier is influenced by changes in intestinal permeability, mucin composition, and homeostasis between the production of new enterocytes and the rate of apoptosis of damaged enterocytes. Diseases such as IBD and colon cancer may lead to disruption of the gut barrier [34]. Certain probiotics have the potential to modify gut integrity [35,36,37].

#### 2.3.1. Tight Junctions

Intestinal barrier function is modulated by tight junction (TJ) proteins such as occludin, zonula occludens-1, claudin-1, claudin-2, claudin-4, junction adhesion molecule-A and F-actin [37,38]. These TJ proteins are located at the sub-apical aspect of the lateral membranes and build the physical connections between cells responsible for integrity of the intestinal barrier [37]. However, intestinal mucosal barrier dysfunction, in conditions such as inflammatory bowel disease, may lead to increased intestinal permeability, which partly results from irregular expression of certain TJ proteins in the intestinal epithelium [36,37]. On the other hand, several studies have revealed the capacity for probiotics to maintain intestinal barrier function by modifying the expression of TJ proteins. The probiotic combination, VSL#3 has been demonstrated to regulate the epithelial TJ protein, occludin, and to decrease the expression of claudin-2, subsequently attenuating increased gut permeability in mice with experimentally-induced Crohn’s disease [36]. Similarly, oral administration of *Lactobacillus plantarum *CGMCC No. 1258 enhanced the expression of TJ proteins such as occludin, ZO-1, claudin-1, claudin-4 and JAM-A in the intestinal epithelium in both the common bile duct and in bile duct ligation challenged rats [37]. In the same study, a relationship was described between protein kinase C (PKC) activity and the ability to redistribute TJ proteins in response to *L. plantarum* administration in rats with experimental obstructive jaundice [37]. In addition, these authors concluded that the protective ability of *L. plantarum* on gut barrier was associated with a decrease in intestinal epithelial cell apoptosis and a reduction of oxidative stress as evidenced by levels of glutathione, malondialdehyde and superoxide dismutase levels in the ileum [37].

#### 2.3.2. Mucin Expression

Integrity of the intestinal microbiota is partly maintained by the structure of mucins [39]. Intestinal mucins, the major protein component of mucus coating the epithelium of the gastrointestinal tract, are highly glycosylated macromolecules produced by epithelial tissues in most metazoans [40,41]. Goblet cells of the enteric epithelium synthesize, store, and secrete mucins to maintain mucus integrity [42]. Moreover, mucins form gels and generate a protective mucus blanket overlying the epithelial surface to protect the mucosa from bacterial overgrowth [42]. Mucins further create an enormous repertoire of potential binding sites for microorganisms [40]. Moreover, it has been reported that certain *Bifidobacterium bifidum* strains can break down glycoprotein linkages within the mucin and facilitate colonocyte contact [25]. Hooper *et al.* elucidated the importance of endogenous microbiota such as *Bacteroides thetaiotaomicron* in assisting the host to degrade indigestible nutrients [43]. This organism degraded certain undigested polysaccharides, undigested starch and host-derived glycans, such as mucins and glycosphingolipids, to monosaccharides in the colon. These monosaccharides are further fermented to result in the production of short chain fatty acids which are utilized by the host as a source of energy [43].

Luminal toxins and alterations in the intestinal microbiota can cause changes in both goblet cell function and the chemical composition of intestinal mucus [44]. In addition, over expression of mucin proteins such as MUC1 has been associated with human cancers [45]. Moreover, chemotherapy agents affect mucins and goblet cell secretions. For example, the chemotherapy agent, 5-FU, has been reported to decrease goblet cell numbers and increase cavitated goblet cells in rats [39].

Certain probiotics have the ability to restore intestinal mucin levels. The structural and functional properties of mucins influence bacterial adhesion activities. The monosaccharide components of mucin carbohydrate side-chains such as *N*-acetyl-glucosamine (GalNac), galactose (Gal), fucose (Fuc), and *N*-acetyl-neuraminic acid (NeuAc) and their ester sulfates, play an important role in the modulation of bacterial adhesion [35,46]. In a recently study, the effects of different concentrations of probiotics (10^8^, 10^9^ and 10^10^ colony forming units of probiotic/kg of soybean meal basal (BD) diets) were investigated on the composition of intestinal mucin monosaccharides and mucosal morphology in broilers [35]. These authors used a probiotic mixture of *Lactobacillus reuteri*, *Enterococcus faecium*, *Bifidobacterium animalis*, *Pediococcus acidilactici* and *Lactobacillus salivarius* that was able to modulate intestinal mucin monosaccharide composition in broilers. Mannose levels decreased linearly with increased probiotic levels in the duodenum; and GalNac and Gal levels also decreased, whereas Fuc levels increased with increasing probiotic intake in the cecum of 42-day-old broilers [35]. These changes in mucin monosaccharide composition may have resulted from the effects of combinations of the different tested probiotics. It is therefore possible that each probiotic strain altered mucin monosaccharide composition differentially. In addition, the probiotic mixture increased villus height and crypt depth, as well as increasing mucin layer thickness in the duodenum in both 14 day and 42-day-old broilers [35]. Probiotics can therefore affect the mucosal barrier and influence gut microbiota composition and bacterial binding activity through adjusting intestinal mucin monosaccharide composition, mucus layer thickness, and intestinal morphology. 

The probiotic *Lactobacillus salivarius* strain UCC118 has been reported to modulate intestinal cell mucin gene production with reduced MUC3A, MUC5AC, and MUC12 gene expression following exposure of the UCC118 mutant (lacking sortase-dependent proteins) to Caco-2 cells [31]. Disruption of the UCC118 sortase gene srtA decreased the adhesion of bacterial to epithelial cells. It was concluded that expression of these mucin genes plays an important role in bacterial adhesion [31]. Recently, Hudcovic *et al.* reported that *Clostridium tyrobutyricum*, administered 7 days before and after induction of colitis by dextran sulphate sodium (DSS) treatment in mice, significantly restored MUC-2 mucin depression induced by DSS [34]. Administration of *Lactobacillus plantarum* Lp91 to healthy mice also significantly up-regulated MUC2 gene expression [47]. 

In general terms, many probiotics have the potential to normalize intestinal integrity through restoration of the mucus layer. This property could be therapeutically important when treating a broad range of intestinal disorders and diseases characterized by mucosal injury.

### 2.4. Influence on Cell Kinetics

Certain probiotics are able to modulate cell kinetics through effects on cell proliferation and apoptosis. This is important in the homeostasis of cell death and reproduction. Especially, the ability of certain probiotics to promote normal cell propagation and concurrently inhibit abnormal cell apoptosis could hold potential in the treatment of certain diseases such as cancer. 

#### 2.4.1. Cell Proliferation

Probiotics may enhance cell proliferation by inhibiting the activity of normal cell apoptosis and also by promoting cell differentiation and cytoprotective activities. Lin *et al.* demonstrated the anti-apoptotic and cytoprotective properties of LGG, *in vivo* and *in vitro*, following challenge with the pro-apoptotic agent, Staurosporine (STS) [48]. LGG pretreatment decreased apoptosis in STS-induced intestinal epithelial IEC-6 cells and Caco-2 cells by significantly reducing terminal deoxynucleotidyl transferase (TUNEL) positivity. Moreover, LGG also inhibited intestinal epithelial apoptosis in 2-week old preweaned mice affected by STS. More importantly, it has been found that the inhibition of caspase 3 activity and regulation of anti-apoptotic genes largely contributed to the anti-apoptotic properties of LGG [48]. Further investigations demonstrated that LGG modulated apoptosis-related genes in a different manner compared to pathogenic *Salmonella typhimurium * [48,49]. LGG has been reported to regulate cellular proliferation and migration and mitogen-activated protein kinase (MAPK) pathways, which play a pivotal role in cell proliferation, differentiation and cytoprotection activities [48,50]. Furthermore, LGG was also reported to induce anti-apoptotic gene transcription without up-regulation of proinflammatory genes, whereas *S. typhimurium* up-regulated anti-apoptotic genes by NF-κB activation [49]. Similar results recently reported by Yanagihara *et al.* illustrated that the expression of genes related to cell proliferation was amplified after exposure of human gastrointestinal epithelial Caco-2 cells to the probiotic *Lactobacillus acidophilus *92 for 20 h [51]. During the same treatment, it was also found that cell proliferation and gene regulation were associated with MAP kinase linked to changes in gene expression for the G-protein coupled receptor, cytochrome P450 and zinc finger protein [51]. 

#### 2.4.2. Cell Apoptosis

Apoptosis is a cell suicide mechanism to control cell numbers in tissues and eliminate individual cells [52]. However, unscheduled apoptosis of certain cells can be detrimental [52]. An increase in the apoptosis to proliferation ratio leads to bacterial invasion and toxin delivery in several diseases. Probiotics have the ability to prevent inflammation-induced cell apoptosis [53]. Yan and Polk reported that LGG was able to prevent cytokines such as TNF-α, IL-1α or γ-interferon inducing apoptosis in both mouse and human colonic epithelial cells [54]. The inhibitory effects of LGG on apoptosis were thought to be due to activation of the anti-apoptotic Akt/protein kinase B, and pro-apoptotic p38/mitogen-activated protein kinase [54]. Moreover, the probiotic strain of *Saccharomyces boulardii* prevented TNF-α induced apoptosis in enterohemorrhagic *Escherichia coli* infected human colonic T84 cells [55]. In the presence of *S. boulardii*, *E. coli* infected cells did not initiate the activation of some apoptotic features such as procaspase-3, poly-ADP-ribose polymerase (PAPR) and internucleosomal cleavage of DNA. Additionally in the same study, *S. boulardii* prevented the activation of caspases-9 and -8, which together led to the anti-apoptotic activities of *S. boulardii* against pathogens [55]. Similar results were obtained by Wang *et al.* who reported that the probiotic mixture, kefir, reduced ovalbumin induced apoptosis in mouse heart tissues by limiting levels of the pro-apoptotic proteins Bax and Bad, cytochrome c and caspase-3 [56]. Administration of probiotic mixtures also depressed expression of apoptosis linked proteins such as pro-apoptotic Bax, caspase-3 and -8, and anti-apoptotic Bcl-2 in the livers of rats fed a methionine choline-deficient diet [57]. The ability of probiotics to modulate apoptotic and anti-apoptotic proteins has also been illustrated in acetaminophen-induced hepatotoxicity [58] and chemotherapy-induced small and large intestinal apoptosis in rats [59]. Furthermore, *Clostridium butyricum* can degrade non-digestible high amylose maize starch to butyrate and acetate. Butyrate (short-chain fatty acid), has been reported to inhibit carcinogenesis in the colon [60]. 

Probiotics can therefore not only induce cell proliferation related gene expression to stimulate cell growth, but also modulate the apoptotic/anti-apoptotic proteins contributing to efficacy in response to cytokine mediated inflammation and apoptosis. 

### 2.5. Immunity

Approximately 70% of the immune system is situated along the intestinal tract as GALT (gut-associated lymphoid tissue); a crucial component of the immune system [61]. A relationship between the intestinal microbiota and host immunity has been widely investigated [3,4,62,63] playing an important role in maintaining host immunity through activation of the immune response during periods of stress, for example against pathogenic threats [6,61,64]. Dysbiosis of the endogenous microbiota can lead to compromised immune responses and contribute to the manifestation of diabetes and other autoimmune diseases [65]. Probiotic administration could be a worthwhile strategy to modulate a disordered intestinal ecosystem. Administration of certain probiotics can enhance immunity [13]. For instance, Moro-Garcia *et al.* reported that dietary supplementation with *Lactobacillus delbrueckii* subsp. *bulgaricus* 8481 for 6 months enhanced the immune response in elderly people, by increasing numbers of circulating NK cells and immature T cell subsets [13]. In addition, the immune risk phenotype (IRP), characterized by an inverted CD4/CD8 ratio, an increase of CD8 + CD28^null^ T cells, and cytomegalovirus (CMV) infection, was also counteracted by this probiotic in a group of elderly people [13,66]. The mechanisms underlying probiotic effects on immunity will be discussed in the following sections.

#### 2.5.1. Immunoglobulin Responses

Immunoglobulin A (IgA) is a major antibody that is secreted across intestinal mucosal linings, playing a critical role in mucosal immunity [67,68]. During pathogenic invasion, low numbers of commensal bacteria remain in intestinal Dendritic cells (DCs) for several days [67]. These commensal-loaded DCs are limited by the mesenteric lymph nodes (MLNs), and repeated intestinal commensal priming results in the production of IgA, selectively and locally, which is responsible for forming one layer of the mucosal barrier against bacterial penetration [67]. 

To date, several studies, have demonstrated the effect of probiotic intake on improved host immunity via the production of IgA [21,69]. Qiu *et al.* in 2012 reported that one-day old broilers fed a diet containing *Lactobacillus casei*, *Bifidobacterium bifidium*, and *Enterococcus faecium* exhibited a more rapid rate of serum antigen specific IgG production and an increase in total IgA in the jejunum than those fed a control diet [69]. Yang *et al.* also reported enhanced serum levels of IgA, IgM and IgG in broiler chickens after being fed the probiotic *Clostridium butyricum *for 40 days [12]. More promisingly, serum levels of IgA, IgG and IgM of colorectal cancer patients who had undergone elective laparoscopic radical surgery, significantly increased after probiotic (Jinshuangqi Tablets, Inner Mongolia Shuangqi Pharmaceutical Co. Ltd., Hohhot, China), administration for 7 days [21]. In the same study, serum IL-2 and CD4^+^ levels also significantly improved [21]. Together, these results reveal that candidate probiotics may improve the immune function of the host via modulation of antigen-specific antibodies. 

#### 2.5.2. Inflammation

A variety of probiotics, such as *Lactobacillus *spp *.*, *Enterococcus faecium* JWS 833 and *Faecalibacterium prausnitzii* [70,71,72] have been reported to maintain immune homeostasis through modulating inflammation in DSS-induced colitis [34], alcohol-induced inflammation [70,73], cytokine-mediated gastrointestinal diseases [74], tumor bearing, and chemotherapy-induced mucositis *in vivo*, as well as pathogen infections *in vitro* [75]. For example, the probiotic *Lactobacillus plantarum* NCC1107 has been reported to reduce lung inflammation in mice by decreasing inflammatory cell numbers, eotaxin and IL-5 [76]. Moreover, dietary delivery of the probiotic *Lactobacillus casei* improved the immune response of mice bearing invasive ductal carcinomas, through significantly increasing production of IL-12 and IFN-γ and increasing NK cell cytotoxicity in spleen cell cultures, and most importantly, reduced tumor growth rate [77]. Furthermore, administration of *Clostridium tyrobutyricum* depressed expression of TNF-α and IL-18 in the descending colon of DSS-treated mice [34]. *Lactobacillus acidophilus* A4 and its cell extracts have been shown to significantly decrease mRNA levels of IL-8, IL-1β, and TNF-α in pathogenic EHEC O157:H7 affected HT-29 intestinal epithelial cells in *in vitro* studies [78]. Recently, Lee *et al.* reported that the probiotic *Lactobacillus rhamnosus* GG attenuated lipopolysaccharide (LPS) induced inflammation of HT-29 cells by blocking TNF-α, and LPS induced IL-8 activation [79]. In *in vitro* studies, Lee *et al.* observed probiotic-induced down-regulation of the inflammatory pathway induced by LPS, including effects on NF-κB nuclear translocation, IkBa degradation and TLR4 mRNA [79]. Similarly, Wang *et al.* recently proposed that inflammation signaling induced by ovalbumin-affected allergy could be involved in increased TLR4 and subsequent activation of phospholate-Jun-*N*-terminal kinase (p-JNK), JNK1/2 (p-NFkB), p-IkB and TNF-α in the hearts of allergy-prone mice [56]. In contrast, a probiotic mixture composed mainly of lactic acid bacteria reversed this inflammation by restricting inflammatory signaling pathways [56]. 

#### 2.5.3. Dendritic Cells and Other Host Immune Responses

Dendritic cells (DCs) are professional antigen-presenting cells, which normally remain in an immature stage in peripheral tissues [80,81]. They can be activated by contact with an antigen, such as a bacterium, resulting in initiation of the maturation process, and subsequently functional changes to the DCs [81]. Furthermore, DCs have an important role in regulating innate and adaptive immune responses by producing cytokines and chemokines [82]. Thus, it is worthwhile to investigate pathways of probiotics and pathogens in stimulating DC maturation in relation to cytokine and chemokine secretion. 

Commensal bacteria, such as *Lactobacillus rhamnosus*, and pathogenic bacteria, such as *Streptococcus pyogenes*, express similar molecular patterns [81]. However, they both induce immune responses through DC maturation and production of type I T helper cells (Th1) cytokines and chemokines in a different way [81]. Indeed, pathogenic *S. pyogenes* was able to induce TNF-α, IL-2, IL-12, IL-23, IL-27, CCL5, CCL19, CCL20, CXCL9 and CXGL10 in human monocyte-derived DCs indicating that this pathogenic bacterium is likely to create cytokine and chemokine environments that polarize the adaptive immune response toward Th1 type and induce inflammation [81]. In contrast, *Lactobacillus rhamnosus* induced a cytokine and chemokine (TNF-α and CCL20) response in DCs, which was significantly lower than *S. pyogenes*. Additionally, *L. rhamnosus* was unable to induce expression of inflammatory cytokines such as IL-2, IL-12, IL-23 or IL-27. Together, these results suggest that DCs have the capacity to distinguish pathogenic and non-pathogenic bacteria and respond to them differently, which in turn may result in the development of distinct adaptive immune responses [81]. 

Certain probiotics have the capacity to stimulate DCs to produce anti-inflammatory cytokines while decreasing levels of pro-inflammatory cytokines induced by pathogens. A new probiotic strain, *Lactobacillus paracasei* CNCM I-4034 isolated from feces of breast-fed newborn infants, secretes bacterial compounds which can be identified by innate pattern-recognition receptors (PRRs), in particular through TLR signaling [83]. *L. paracasei* CNCM I-4034 also reduced enteropathogenic (*Salmonella typhi*)-induced pro-inflammatory cytokine (IL-8, IL-6 and TNF-α) and chemokine (MCP-1, CCL2, RANTES and CCL5) production. Furthermore, *L. paracasei* CNCM I-4034 increased the secretion of the anti-inflammatory cytokine, transforming growth factor beta (TGF-β_2_), in human DCs, contributing to a reduction of inflammation [83]. *Bifidobacterium breve* UCC2003 produces a cell-surface-associated exopolysaccharide (EPS), linked to the evasion of adaptive B-cell responses, and believed to facilitate various aspects of a commensal-host interaction, as well as reduced colonization levels of the gut pathogen *Citrobacter rodentium in vivo* [84]. O’Callaghan *et al.* reported that *L. salivarius* UCC118 has the capacity to stimulate the expression of genes, such as CCL20, CXCL1, CXCL2, TNFAIP3, NFKBIA and BIRC3 in contact with Caco-2 cells. These genes are able to depress inflammation via down-regulating NF-κB and reducing cytokine-induced apoptosis [31].

#### 2.5.4. Inflammasomes

In a recent commentary, Howarth proposed that probiotics/prebiotics could modulate intestinal inflammation, immune response and function through an effect on inflammasomes [85]. Inflammasomes are a group of protein complexes built around several proteins including NLRP3, NLRC4, AIM2 and NLRP6 [86]. Inflammasomes can identify a wide range of microbial, stress and damage signals, and following the activation of capase-1 subsequently induce the secretion of pro-inflammatory cytokines such as IL-1 and IL-18 [86]. Miettinen *et al.* reported that nonpathogenic *Lactobacillus rhamnosus* could activate inflammasome functions and enhance antiviral activity in human macrophages [87]. Two *L. rhamnosus* strains, LGG and LC705, have been demonstrated to activate inflammasomes and as a result, stimulate levels of IL-1β in macrophages which are essential for caspase-1 activity [87]. However, it is unclear which inflammasomes are involved in the stimulation of IL-1β secretion [87]. Furthermore, in the same study, LC705 showed antiviral activity by inducing type 1 interferon-gene activation, associated with influenza A virus replication and viral protein production in macrophages [87]. However, in contrast, Strowing *et al.* concluded that inflammasomes can induce pyroptosis, a form of cell death, whilst inflammasome-mediated processes play an important role in microbial infections, regulation of metabolic processes, and mucosal immune responses in human diseases [86]. Indeed, a recent study by Qu *et al.* found that inflammasome NLRC4 phosphorylation (NLRC4 phospho-Ser 533) successfully inhibited the activation of caspase-1 and pyroptosis in response to *Salmonella typhimurium* infection [88]. NLRC4 phosphorylation could therefore be a decisive point for NLRC4 inflammasome activation and host innate immunity [88]. 

Further clarification of the role of inflammasomes and their modulation of intestinal inflammation, immune responses and gut function is required before we can understand the impact of probiotics and prebiotics on inflammasome-mediated processes. 

### 2.6. Safety

Probiotics are commonly considered to be human-friendly and non-pathogenic bacteria. However, an awareness of safety implications should be maintained. Recent studies have found that the probiotic *Escherichia coli* Nissle 1917, a promising candidate for therapy against mucosal disorders [89,90], can induce DNA damage *in vivo* and trigger genomic instability and gene mutations in mammalian cells [91,92]. This damage is involved in the development of colorectal cancer [91]. Furthermore, it has been reported that in the genome of *E. coli* Nissle 1917, a cluster of genes called “*pks*” island plays an important role in producing a hybrid peptide polyketide genotoxin, termed colibactin, which is responsible for genetic damage [91,92,93]. However, Olier *et al.* recently reported that the probiotic activity of *E. coli* Nissle 1917 should not be abolished by its genotoxic activity [14]. In their study, *E. coli* Nissle 1917 effectively reduced the colonic damage caused by DSS, with a decreased colitis score, decreased myeloperoxidase activity and pro-inflammatory cytokine IL-1β levels and increased IL-10 levels. However, *E. coli* Nissle 1917 has also been demonstrated to induce high levels of DNA double strand breaks in cultured intestinal crypt cells [14]. In another set of experiments Olier *et al.* tested the effect of an isogenic mutant of *E. coli* Nissle 1917 (Nissle *ΔclbA*) on DSS-induced colitis in rats and reported a disruption of the *clbA* gene and disabled activity of the *pks* island [14]. The Nissle *ΔclbA* mutant was unable to induce the same damage as wild type *E. coli *Nissle 1917. However, at the same time, the Nissle *ΔclbA* mutant also lost its probiotic function and did not have the capacity to reduce the severity of DSS-induced colitis, and in some cases, even exacerbated the damage [14]. Therefore, these results demonstrate that colibactin (from wild type *E. coli* Nissle 1917) could induce damage, but it may also play a pivotal role in the efficacy of *E. coli* Nissle 1917. Moreover, it is hypothesized that colibactin possesses anti-inflammatory activity and immunomodulatory functions. In addition, colibactin may consist of more than one molecule, which could be encoded by the biosynthetic gene clusters of the *pks* island, leading to its probiotic activity in the gut [14]. 

Different strains of the same probiotic may also impart opposite effects, and results obtained from animal models could very well differ from those obtained in human studies. Further research is required to better identify the benefits and potential risks of probiotic administration.

## 3. Probiotic-Derived Factors

Overall, interest in investigating the impact of probiotics has grown steadily during the past decade, with most studies focusing on the mechanisms and clinical applications of probiotics associated with intestinal disorders *in vivo* and *in vitro* [17,18,94,95]. In contrast, the beneficial efficacy of probiotic-derived factors has been less documented, and only in recent years has there been an upsurge in research into the properties of probiotic-derived factors [74,96]. Further investigation of these factors could be used to achieve therapeutic benefits whilst avoiding risks related to the administration of live bacteria [20]. In addition, these factors hold potential for the development of safer therapeutic medicines, and provide a better understanding of the underlying mechanism of probiotics. 

### 3.1. Competition with Pathogens

Several studies have revealed the capacity for probiotics to secrete probiotic factors, such as bacteriocins and reuterin, which have been shown to inhibit the adhesion and viability of known enteric pathogens [20,97]. These factors could be a rich source of new anti-pathogenic compounds that may play an important role in restricting the activities of pathogens. *Lactobacillus reuteri* formed biofilms that produced antimicrobial glycerol derivatives referred to as reuterin, which is a potent anti-pathogenic compound, and has been shown to inhibit a wide range of microorganisms [97,98]. Recently, *Lactobacillus acidophilus* ATCC 4356 has been reported to produce a proteinaceous molecule which demonstrated inhibitory activity against eight of the human *Campylobacter jejuni* strains [99]. This suggests that *Lactobacillus acidophilus* ATCC 4356 has the potential to act as an antimicrobial agent for the treatment of *Campylobacter* infections in humans [99].

Moreover, Fayol-Messaoudi *et al.* reported that the probiotic strains *Lactobacillus johnsonii* La1, *Lactobacillus rhamnosus* GG (LGG), *Lactobacillus casei* shirota YIT9029, *L. casei* DN-114 001, and *L. rhamnosus* GR1 produced non-lactic acid molecules in their cell-free cultured supernatants [100]. These non-lactic acid molecules were believed to be responsible for killing activity against pathogenic *Salmonella enterica* serovar Typhimurium SL1344 [100]. During the same study, it was found that the reduction of pH in the culture media could have contributed to the restriction of Serovar typhimurium SL1344 growth *in vitro* [100]. It is equally important to note that, *Lactobacillus johnsonii* NCC 533 isolated from human intestines, as well as eight different *L. johnsonii* strains and *Lactobacillus gasseri* were able to produce H_2_O_2_, under the conditions used to incubate these cells, and also in the presence of oxygen. More importantly, the supernatants from NCC 533, cultured in the presence of oxygen contained H_2_O_2_, which effectively killed the pathogen *Salmonella enterica* serovar Typhimurium SL1344 *in vitro * [101].

More recently, Gomes *et al.* found that *Lactobacillus sakei* 1 produced a heat-stable antimicrobial peptide (sakacin 1, a class IIa bacteriocin) in its culture supernatant [102]. This cell-free supernatant containing sakacin 1 exhibited the ability to reduce the chance of infection by the pathogen *Listeria monocytogenes *in human intestinal Caco-2 cells [102]. This ability is consistent with the inhibitory properties of probiotic *Lactobacillus plantarum* strain LP 31, which can also produce a bacteriocin that inhibits growth of pathogenic bacteria such as *Pseudomonas* spp., *Staphylococcus aureus*, *Bacillus cereus* and *Listeria monocytogenes* [103]. Moreover, supernatants from *Bifidobacterium breve* 46 and *Bifidobacterium lactis* 8:8 inhibited the growth and toxin production of *Clostridium difficile* (CD) NAP1/027 strain [104]. Seo *et al.* recently reported that the probiotic EcN can produce an antimicrobial defensin-mature fusion beta-defensin 2 (HisMHBD2) derivative in a soluble form [29]. This HisMHBD2 protein has demonstrated antimicrobial activity against the growth of *Escherichia coli* K-12 MG1655, *Salmonella enterica* serovar Typhimurium SL1344 and *Listeria monocytogenes* EGD. In addition, the recombinant EcN strain encoded a fusion protein containing YeBF gene, and the mature part of HisMHBD2 resulting in the production of YebFMHBD2, which also exhibited a significant inhibition of the growth of *E. coli*, *Salmonella* and *L. monocytogenes* [29]. These results indicate that EcN-released proteins could exert antimicrobial efficacy against several pathogenic species.

Combined, these derived factors, released from live probiotics, and found in their culture supernatants could have activities similar to their original “parent” probiotics. Supernatants from certain probiotics could potentially lower the likelihood of infection in the host and indirectly assist in maintaining intestinal integrity and immunity.

### 3.2. Maintenance of Intestinal Integrity

The improvement of intestinal barrier function can be modulated by certain probiotic species. For example, certain probiotics can decrease gut permeability, through strengthening of the gut barrier against pathogen invasion. Moreover, the gut barrier can also be influenced by modulation of the mucus layer and/or tight junctions [20]. In an *in vitro* study, Wang *et al.* recently reported that LGG supernatants prevented alcohol-induced Caco-2 cell monolayer barrier dysfunction by ameliorating alcohol-induced decreases in epithelial cell resistance and increases in permeability [105]. 

More recently, Wang *et al.* have reported the effects of LGG supernatant administration on alcohol-induced intestinal barrier and liver damage in mice [73]. The LGG supernatant restored the alcohol-induced reduction in ileum mRNA levels of claudin-1, thereby showing the capacity for LGG-released factors to alter tight junction proteins [73]. Moreover, during an alcohol challenge, the intestinal epithelial cells experienced an oxygen adaption process, characterized by the expression of a master transcription factor, referred to as hypoxia inducible factor (HIF). This HIF is involved in inducing mucin production, regulation of intestinal trefoil factor (ITF), and the activities of P-glycoproten (P-gp) and other nucleotide signaling, which together play an important role in intestinal integrity [73,106,107]. Meanwhile, LGG supernatant pretreatment has demonstrated the ability to modulate mRNA levels of ITF, P-gp and cathelin-related antimicrobial peptide (CRAMP) in the ileum area of alcohol-challenged mice to partially restore intestinal barrier function [73]. 

Protein A20 expressed in human colon epithelial cell lines (HT-29) is crucial for the degradation of endocytic allergens by facilitating endosome/lysosome fusion, which in turn maintains the gut epithelial barrier [95]. Probiotic (*Clostridium butyricum* CGMCC0313-1)-derived proteins extracted from the culture supernatants were able to enhance expression of A20 in HT-29 cells, resulting in an enhanced barrier function [95]. 

A number of studies have reported the mechanism of live probiotics acting on the intestinal barrier by modulating tight-junction proteins and mucin levels *in vivo* and *in vitro* [22,25,35]. Evidence is still lacking for probiotic-derived factors to modulate the gut barrier in this context, although studies in *in vitro* models are underway. 

### 3.3. Cell Kinetics

Several attempts have been made to investigate the effects of probiotic-derived factors on apoptosis *in vitro* [74,108,109]. A recent study by Prisciandaro *et al.* reported that supernatants from EcN and LGG significantly lowered the caspase activity of 5-FU challenged IEC-6 cell lines, suggesting their potential ability to prevent or inhibit enterocyte apoptosis induced by 5-FU [108]. The anti-apoptosis properties of probiotic LGG supernatants perhaps be explained by Yan *et al.* who identified 2 novel proteins, p75 (75 kDa) and p40 (40 kDa), both of which have been shown to play pivotal roles in reducing TNF-α-induced epithelial cell (Human and Mouse colon epithelial cells and cultured colon explants) apoptosis and importantly, promote the growth of these epithelial cells [74]. Moreover, p75 and p40 were also found to be responsible for Akt activation by LGG released factors [74]. Together, these results suggest a promising application for the prevention of cytokine-mediated gastrointestinal diseases by using LGG-derived proteins. 

A recent study by Lebeer *et al.* further specified the functions of p75, renamed as Msp1, secreted by LGG [110]. These investigators reported that Msp1 is an *O*-glycosylated protein which can be glycosylated with mannose-specific Concanavalin A (ConA) reactive sugars at serine residues of 106 and 107. Glycosylation for Msp1 is hypothesized to be involved in various glycan-mediated interactions of LGG, such as targeting specific innate immune cells and food degradation by microbes [110,111]. The investigation of protein glycosylation in probiotics could play an important role in understanding the signaling process between the microbe and host, as these signaling pathways can be mediated by glycans through specific protein glycosylation [110]. This study suggested that a species-specific glycosylation mechanism for Msp1 could occur in different *L. rhamnosus* bacteria, which implies that the released factors from other probiotics could be glycosylated at other sites with other sugars. Additional research is required to thoroughly characterize the *O*-glycans and exact glycosylation sites of Msp1 [110] and also to identify other possible glycosylated proteins released from probiotics and their effects on glycan-mediated signaling pathways in response to disease or under normal conditions.

### 3.4. Immunity

#### 3.4.1. Inflammation

In recent years the number of studies investigating the effects of probiotic-derived factors on pathogen-induced or oxidative stimuli-induced inflammation has increased. For example, *Lactobacillus reuteri*-formed biofilms have the ability to suppress human TNF production in LPS-activated monocytoid cells [98]. In addition, *Bifidobacterium breve* and *Streptococcus thermophilus* have the ability to release metabolites (<3000 Da) which inhibit TNF-α secretion from lipopolysaccharide (LPS) affected peripheral blood mononuclear cells or the THP-1 cell line [75]. In addition, Menard *et al.* suggested that this anti-inflammatory effect (anti-TNF-α) was partially a result of the ability of *B. breve* and *S. thermophilus* released factors to suppress LPS-FITC (a fluorescent marker for detection by flow cytometry) binding to THP-1 cells, and also to inhibit NFκB activation [75]. Moreover, Caco-2 cells pre-treated with spent culture supernatants of *Lactobacillus plantarum* 2142 for one hour were able to inhibit the growth of *Salmonella enteritidis* 857 [112]. In this study it was also found that spent culture supernatants decreased levels of IL-8 synthesis, and in addition, induced the expression of Heat-shock protein (Hsp) 70 in *S. enteritidis* 857 infected Caco-2 cells [112]. These results demonstrated that *L. plantarum* 2142-released factors could exert anti-inflammatory effects by depressing IL-8 secretion and indirectly increasing levels of Hsp70 [112]. Recently, Paszti-Gere *et al.* reported the ability of *L. plantarum* 2142 SCS to significantly decrease pro-inflammatory IL-8 and TNF-α levels in porcine IPEC-J2 enterocytes in response to oxidative stress (H_2_O_2_) [96]. In addition, during the same experiment, Hsp70 gene expression was also significantly promoted, which demonstrated the importance of Hsp70 in response to oxidative or other stresses [96]. Importantly, *L. plantarum* 2142-specific proteins (21 and 31 kDa molecular weight) identified from spent culture supernatants of *L. plantarum* 2142, could be potential bioactive molecules for modulating inflammation and oxidative stress effected by various stimuli or diseases [96]. Probiotic-derived factors could therefore exert similar effects to their living probiotic counterparts by modulating chemotactic cytokine secretion and reducing inflammation resulting from pathogen invasion or intestinal disorders.

#### 3.4.2. Dendritic Cells

Recently, it has been reported that probiotic *Lactobacillus paracasei* CNCM I-4034 cell free supernatants showed similar effects to live probiotic *Lactobacillus paracasei* CNCM I-4034, at reducing pro-inflammatory TNF-α and chemokine MCP-1 levels in human DCs challenged with enteropathogenic *Salmonella * [83]. These supernatants also acted as potent inducers of anti-inflammatory TGF-β_1_ in reaction to *Salmonella*. It was hypothesized that certain factors released from *L. paracasei* CNCM I-4034, such as bacteriocins, could play an important role [83]. However, the effects were unlikely to be caused by acidic compounds, as the probiotic supernatants were neutralized to pH 7.0 for use [83]. Both living probiotics and pathogens can stimulate DC maturation, resulting in the secretion of cytokines and chemokines [81]. However, it is still unclear whether probiotic-derived factors stimulate DC maturation differently compared to pathogens.

#### 3.4.3. Other Host Immune Responses

In 2012, López *et al.* reported the importance of probiotic bacteria co-culture with epithelial cells. It was found that soluble factors could be secreted in the supernatant of *Bifidobacterium bifidum* LMG13195 after being previously co-cultured with HT29 cells [113]. These soluble factors play an important active immunoregulatory role by enhancing numbers of CD4^+^CD25^high^ cells expressing chemokine receptor Treg markers in human peripheral blood mononuclear cells (PBMCs) [113]. However, the supernatants of Bifidobacterium strains cultured without contact with epithelial cells were unable to exert significant beneficial effects on PBMCs [113,114]. This gives rise to a new concept that the biological impact of probiotic-derived factors could be potentiated if the supernatants from the original live probiotics were previously co-cultured with epithelial cells.

### 3.5. Anti-Carcinogenic Properties

The potential utility of probiotic-derived factors in cancer therapy represents a new frontier. Fatty acids such as butyrate have demonstrated anti-carcinogenic properties and *Clostridium butyricum* produces high levels of butyrate. Cousins *et al.* in 2012 found that *Propionibacterium freudenreichii* ITG P9 strain fermented milk supernatants induced apoptosis of HGT-1 cancer cells in a time and dose dependent manner [109]. The features of apoptosis in cancer cells affected by *P. freudenreichii* fermented milk supernatants included condensed and fragmented chromation, DNA laddering and accumulation of cells in subG1 cell cycle phase, reactive oxygen species accumulation, mitochondrial transmemebrane potential disruption, caspase activation and cytochrome c release [109]. These results indicate that certain probiotic-derived compounds could exert a cytotoxic effect on cancer cells, and may synergistically assist the action of certain chemotherapy drugs, such as camptothecin, to kill cancer cells [109]. The strategy of using these probiotic-derived factors as a food supplement for the patient could potentially reduce the required dose of chemotherapy treatment for cancer. However, this concept requires further investigation.

## 4. Conclusions and Future Directions

Determining the precise composition of secreted products from probiotic bacteria is challenging, and will depend on species, strain, micro-environment and culture conditions. In addition, it is rare for any individual probiotic to act through a single mechanism, and its biological impact is influenced by factors including dose, frequency of administration, and the composition of the microbiota. Given the enormous numbers of bacterial species, strains and sub-strains in the microbiota, the number of potential probiotics, and consequently, sources of probiotic-derived factors, is equally far-reaching. Considered together, future studies adopting a more targeted approach to the identification of probiotic biofactors could reveal a bright future for the clinical application of specific biofactors for a range of digestive disease conditions.
